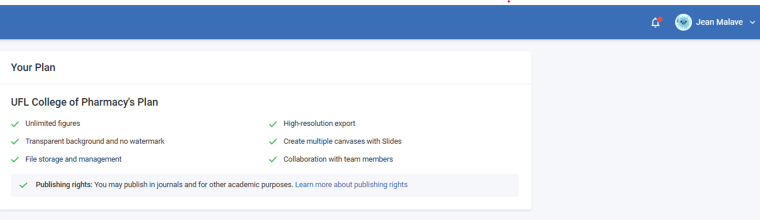# Impact of Area-Level Socioeconomic Deprivation on Post-PCI Outcomes Stratified by P2Y_12_ Inhibitor Therapy

**DOI:** 10.1016/j.jacadv.2025.102577

**Published:** 2026-02-03

**Authors:** Jean G. Malavé, Cameron D. Thomas, Francesco Franchi, Ellen C. Keeley, Caitrin W. McDonough, Luis Ortega-Paz, Danwei Shao, Joseph S. Rossi, George A. Stouffer, Masoud Rouhizadeh, Dominick J. Angiolillo, Craig R. Lee, Larisa H. Cavallari

**Affiliations:** a Department of Pharmacotherapy and Translational Research and Center for Pharmacogenomics and Precision Medicine, College of Pharmacy, University of Florida, Gainesville, Florida, USA; b Division of Cardiology, Department of Medicine, College of Medicine-Jacksonville, University of Florida, Jacksonville, Florida, USA; c Division of Cardiovascular Medicine, College of Medicine, University of Florida, Gainesville, Florida, USA; d Division of Pharmacotherapy and Experimental Therapeutics, Eshelman School of Pharmacy, University of North Carolina at Chapel Hill, Chapel Hill, North Carolina, USA; e Division of Cardiology, School of Medicine, University of North Carolina at Chapel Hill, Chapel Hill, North Carolina, USA; f Department of Pharmaceutical Outcomes and Policy, College of Pharmacy, University of Florida, Gainesville, Florida, USA

**Keywords:** antiplatelet therapy, clopidogrel, CYP2C19, percutaneous coronary intervention, social determinants of health

## Abstract

**Background:**

Social deprivation influences postpercutaneous coronary intervention (PCI) outcomes, but whether it affects outcomes by antiplatelet therapy is unknown.

**Objectives:**

The impact of area-level social determinants of health on the effectiveness and safety of P2Y_12_ inhibitors following PCI was assessed.

**Methods:**

Data were abstracted from electronic health records for adults who underwent PCI and clinical cytochrome P450 2C19 genotyping and received a P2Y_12_ inhibitor across 3 institutions. Social Deprivation Index (SDI) and Social Vulnerability Metric (SVM) were assigned using zip code tabulation areas. Multivariable Cox regression was used to evaluate the association between SDI and SVM (per unit increase) and risk for major atherothrombotic events (MAE) (death, myocardial infarction, stent thrombosis, ischemic stroke, or revascularization for unstable angina) and bleeding (Global Use of Strategies to Open Occluded Coronary Arteries Moderate-Severe) with clopidogrel and alternative therapy (eg, prasugrel or ticagrelor).

**Results:**

Overall, 3,141 patients (mean age: 63 years; 33% female; 68% with acute coronary syndrome) were included. In clopidogrel-treated patients (n = 1,852), SDI was associated with higher risk for MAE (adjusted HR: 1.009; 95% CI: 1.001-1.016; *P* = 0.018) but not bleeding (adjusted HR: 1.001; 95% CI: 0.990-1.012; *P* = 0.838). In patients on alternative therapy (n = 1,289), SDI was associated with a higher risk for bleeding (adjusted HR: 1.014; 95% CI: 1.001-1.028; *P* = 0.048), but not MAE (adjusted HR: 0.998; 95% CI: 0.988-1.007; *P* = 0.652). SVM was not significantly associated with adverse outcomes with clopidogrel or alternative therapy after adjustment.

**Conclusions:**

Higher SDI was associated with an increased risk of MAE with clopidogrel and bleeding with alternative therapy following PCI. Whether addressing socioeconomic disparities improves P2Y_12_ inhibitor-related outcomes remains to be determined.

Coronary artery disease remains a leading driver of cardiovascular mortality in the United States.^1^ Percutaneous coronary intervention (PCI) followed by dual antiplatelet therapy, consisting of aspirin plus a P2Y_12_ inhibitor (ie, clopidogrel, prasugrel, ticagrelor), significantly improves outcomes among patients with chronic coronary disease or acute coronary syndrome (ACS).^2^ The impact of clinical and genetic factors on antiplatelet response, specifically with clopidogrel, and post-PCI outcomes is well documented.^3^, ^4^, ^5^ Clopidogrel requires cytochrome P450 2C19 (CYP2C19)–mediated bioactivation, with those carrying a *CYP2C19* loss-of-function (LOF) variant being at significantly increased risk for treatment failure.^3^^,^^6^ However, even when accounting for *CYP2C19* genotype and other factors known that influence clopidogrel response (eg, age, body size, diabetes, chronic kidney disease, drug-drug interactions, smoking), there remains significant interpatient variability in the safety and effectiveness of clopidogrel.^4^^,^^5^^,^^7^ While prasugrel and ticagrelor demonstrated superior efficacy compared to clopidogrel in clinical trials, they are associated with a higher risk of bleeding, and current bleeding risk-prediction models demonstrate limited discriminatory ability, suggesting that key risk factors remain unaccounted for.^8^, ^9^, ^10^, ^11^ Growing evidence shows that social determinants of health (SDOH) are strong predictors of cardiovascular morbidity and mortality, including among patients post-PCI; although whether their impact varies by antiplatelet therapy has yet to be evaluated.^12^, ^13^, ^14^ Given the variable propensity for bleeding and cardiovascular events among P2Y_12_ inhibitor-treated patients, investigating this association may help inform strategies to address disparities in cardiovascular outcomes.

SDOH are nonclinical factors (eg, housing, income, education) that impact health outcomes.^15^ Composite SDOH scores quantify the combined impact of social, economic, and environmental factors at an area-level.^16^ Two such scores are the Social Deprivation Index (SDI) and Social Vulnerability Metric (SVM).^17^^,^^18^ The SDI, developed using data from the American Community Survey, comprises 7 variables focused on socioeconomic deprivation (eg, income, employment, education, housing, family structure, and vehicle ownership). The SVM, derived from the Agency for Healthcare Research and Quality Database, includes 24 variables stemming from 5 broader domains: demographics, education, economic context, physical infrastructure, and health care. Both scores have been associated with area-level variation in all-cause mortality.^17^^,^^19^^,^^20^

This study evaluated the association between SDI and SVM scores and risk for cardiovascular and bleeding events in patients who underwent PCI and *CYP2C19* genotyping, stratifying by P2Y_12_ inhibitor therapy (ie, clopidogrel vs alternative P2Y_12_ inhibitor therapy) and adjusting for clinical factors well recognized to influence post-PCI outcomes.

## Methods

### Study population

Patients enrolled into the Precision PCI registry (NCT06143709) at the University of Florida, Gainesville; University of Florida, Jacksonville; or University of North Carolina, Chapel Hill with available geographic data were included.^21^ Briefly, the registry includes adult patients who underwent PCI, received clinical *CYP2C19* genotyping, and were prescribed a P2Y_12_ inhibitor. Genotyping-guided recommendations were to avoid clopidogrel and use alternative therapy (ie, prasugrel or ticagrelor) in patients with a LOF (ie, *∗2* or *∗3*) allele in the absence of contraindications. No recommendations were made for those without a LOF allele, and the ultimate prescribing decision was left to the cardiologist’s discretion. Data collection procedures were approved by the University of Florida and University of North Carolina Institutional Review Boards, and written informed consent was obtained from prospectively enrolled participants.

### Data abstraction

Demographic, clinical, and geographic (ie, 5-digit zip code) data were manually abstracted from electronic health records (EHRs) for patients included in this analysis. Data abstraction was performed beginning with the PCI encounter associated with *CYP2C19* genotyping (index PCI) and up to 12 months post-PCI, the occurrence of an outcome of interest, or P2Y_12_ inhibitor discontinuation. For patients enrolled prospectively, EHR data collection procedures were supplemented with phone calls at 1, six, and 12 months post-PCI. SDOH composite scores were sourced from the Robert Graham Center (2016) and Social Vulnerability Metric Data Tables (2018) at a zip code tabulation area (ZCTA) level, with variables in each score listed in Supplemental Table 1.^17^^,^^18^ Score versions were selected based on proximity to the median year of enrollment (2016) and were assigned to each individual based on the ZCTA of residence at the time of the index PCI. Treatment groups (ie, clopidogrel or alternative therapy) were defined based on the P2Y_12_ inhibitor at the time of event or last follow-up, as previously described.^22^^,^^23^

### Study endpoints

The primary outcome was the composite of major atherothrombotic events (MAEs), defined as all-cause-mortality or first occurrence of myocardial infarction, ischemic stroke, stent thrombosis, or unstable angina requiring revascularization. The secondary outcome was clinically significant bleeding, defined as a Global Use of Strategies to Open Occluded Coronary Arteries classification of moderate to severe/life threatening bleeding.^24^ Events were identified via EHR review based on provider-reported diagnoses and through patient interview for those enrolled prospectively. All clinical events were independently verified by an interventional cardiologist or reviewed by a clinical pharmacist.

### Statistical analysis

The Wilcoxon rank sum test was used to compare the distribution of area-level SDOH scores in the study cohort to that of the U.S. population. The national score distribution was determined using ZCTA-level population data from the American community survey 5-year estimate for 2016 to 2020.^25^ Unadjusted event rates (per 100 patient-years [PY]) were reported for the overall population and by P2Y_12_ inhibitor treatment group. Multivariable Cox proportional hazards models were generated to evaluate the association between SDOH composite scores and risk for cardiovascular and bleeding events. SDOH composite scores were evaluated on a continuous scale using raw SDI scores and SVM percentiles. All models were adjusted for age, sex, self-reported race, body mass index, and known risk factors of each outcome. Specifically, for MAE, models were also adjusted for history of hypertension, dyslipidemia, chronic kidney disease, heart failure, atrial fibrillation, diabetes, peripheral vascular disease, stroke, current smoking, prior PCI, and ACS as a PCI indication.^26^ For clinically significant bleeding, models were adjusted for history of chronic kidney disease, diabetes, peripheral artery disease, gastrointestinal or intracranial hemorrhage, dyslipidemia, and anticoagulant use at discharge.^27^ Models evaluated within clopidogrel-treated patients were further adjusted for *CYP2C19* LOF carrier status. Stratification by enrollment site was performed for all models. Proportional hazards assumptions were assessed by plotting scaled Schoenfeld residuals. To assess the robustness of our findings, bleeding was also evaluated using Fine-Gray competing risk models, treating death as a competing event. To illustrate the effect of SDOH on risk, we plotted the median adjusted predicted risk for events across SDOH deciles. Sensitivity analyses for potential nonlinear relationships were conducted by fitting spline-based models and evaluating improvements in model fit using likelihood ratio tests. To contextualize the clinical significance of SDOH, we calculated adjusted 1-year absolute risk difference between extreme decile for scores that demonstrated a significant association in our adjusted Cox model (*P* < 0.05) and compared these to absolute risk difference for established clinical risk factors. All statistical analyses were performed using the R statistical software (version 4.3.2).^28^

## Results

### STUDY population and baseline characteristics

**Figure 1:**
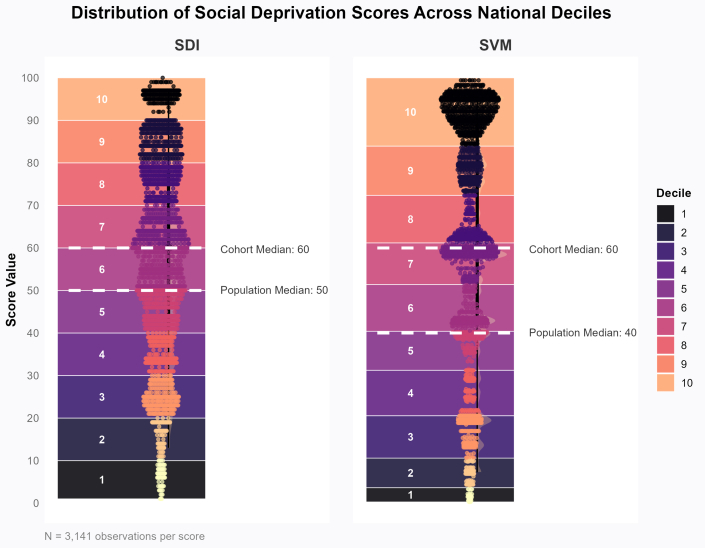
**Distribution of Social Determinants of Health Scores Across the Study and U.S. Populations** Scatter plots show the distribution of Social Deprivation Index and Social Vulnerability Metric values for the study cohort. Behind the points, colored horizontal bands represent the national distribution of each score, with each band corresponding to 1 decile of the U.S. population. The vertical width of each band reflects the range of score values within that decile based on zip code tabulation area-level distribution in the American Community Survey (2016). Thus, wider bands indicate deciles where score values are more dispersed, while narrower bands indicated deciles with a tighter range. Dashed lines indicate the median score for the study cohort and the U.S. population. SDI = Social Deprivation Index; SVM = Social Vulnerability Metric.

In total, 3,141 patients (mean age: 63 ± 12 years; 33% female; 68% with an ACS indication for PCI) were included. At the time of event or last follow-up, 1,852 (59%) were treated with clopidogrel, and 1,289 (41%) were on alternative therapy. Baseline characteristics for each treatment group are summarized in Table 1. In the overall cohort, the median (IQR) SDI score was 60 (41-78), and the median (IQR) SVM percentile was 60 (42-89). No significant differences in SDI (60 [41-78] vs60 [41-79], *P* = 0.400) or SVM (62 [42-89] vs59 [40-89], *P* = 0.091) were observed between clopidogrel and alternative P2Y_12_ inhibitor treatment groups, respectively. Across study sites, median SDI scores were 60 (43-76) for Gainesville, 60 (41-79) for Jacksonville, and 61 (40-81) for Chapel Hill (*P* = 0.723). The median SVM percentiles were 76 (44-90) for Gainesville, 58 (40-80) for Jacksonville, and 60 (26-89) for Chapel Hill (*P* < 0.001). The distribution of the cohort’s SDOH scores compared to the national distribution is shown in Figure 1. Compared to the median SDI (50 [25-75]) and SVM (40 [16-66]) scores of the U.S. population, our cohort presented with significantly higher SDOH scores (*P* < 0.001 for both comparisons).

**Table 1:** Baseline Characteristics by Treatment Group

Characteristic	Overall Cohort (N = 3,141)	Clopidogrel-Treated Patients (n = 1,852)	Alternative Therapy-Treated Patients^a^ (n = 1,289)
Demographics			
Age (years)	63 ± 12	65 ± 12	61 ± 11
Female	1,023 (33)	617 (33)	406 (31)
Self-reported race			
White	2,396 (76)	1,454 (79)	942 (73)
Black	604 (19)	318 (17)	286 (22)
Other^b^	141 (5)	80 (4)	61 (5)
*CYP2C19* LOF allele carrier	920 (29)	393 (21)	527 (41)
BMI (kg/m^2^)	30 ± 7	30 ± 7	31 ± 6
Current smoker	847 (27)	484 (26)	363 (28)
SDOH composite measures			
SDI score	60 [41-78]	60 [41-78]	60 [41-79]
SVM percentile	60 [42-89]	62 [42-89]	59 [40-89]
Past medical history			
ACS as PCI indication	2,127 (68)	1,177 (64)	950 (74)
Prior PCI	908 (29)	565 (31)	343 (27)
Hypertension	2,607 (83)	1,556 (84)	1,051 (82)
Dyslipidemia	2,189 (70)	1,343 (73)	846 (66)
Diabetes	1,237 (39)	751 (41)	486 (38)
Myocardial infarction	799 (25)	509 (27)	290 (22)
Stroke or TIA	381 (12)	267 (14)	114 (9)
PVD	285 (9)	194 (10)	91 (7)
Heart failure	552 (18)	388 (21)	164 (13)
Atrial fibrillation	286 (9)	236 (13)	50 (4)
Gastrointestinal hemorrhage	81 (3)	56 (3)	25 (2)
Intracranial hemorrhage	14 (0.4)	12 (0.6)	2 (0.2)
Cancer	154 (5)	116 (6.2)	38 (2.9)
Chronic kidney disease	725 (23)	490 (26)	235 (18)
Discharge medications			
Aspirin	3,015 (96)	1,762 (95)	1,253 (97)
Oral anticoagulation	337 (11)	281 (15)	56 (4)
ACEI or ARB	2,122 (68)	1,207 (65)	915 (71)
Beta-blocker	2,567 (82)	1,512 (82)	1,055 (82)
Statin	2,950 (94)	1,717 (93)	1,233 (96)

Value are n (%), mean ± SD, or median [IQR].

ACEI = angiotensin-converting enzyme inhibitor; ACS = acute coronary syndrome; ARB = angiotensin receptor blocker; BMI = body mass index; CYP2C19 = cytochrome P450 2C19; LOF = loss-of-function; PCI = percutaneous coronary intervention; PVD = peripheral vascular disease; SDOH = social determinants of health; SDI = social deprivation index; SVM = social vulnerability metric; TIA = transient ischemic attack.

a Alternative therapy consisted of: prasugrel (n = 754; 58.5%), ticagrelor (n = 531; 41%), cangrelor (n = 2; 0.2%), and high-dose clopidogrel (n = 2; 0.2%).

b Patients classified as a self-reported race of “other” consisted of: Asian, Native Hawaiian or other pacific islander, American Indian or Alaska Native, mixed race; or unknown.

### SDOH and ischemic outcomes

**Figure 2:**
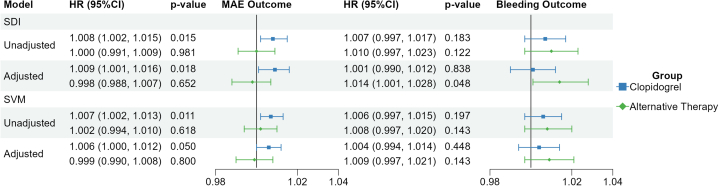
**Association of Social Determinants of Health Composite Scores With Outcomes by Treatment Group** Associations were evaluated using Cox proportional hazards models. All models were adjusted for established clinical risk factors and stratified by enrollment site. Scaled Schoenfeld residuals revealed no violations to the proportional hazards’ assumptions. SDI = Social Deprivation Index; SVM = Social Vulnerability Metric; MAE = major adverse cardiovascular events.

**Figure 3:**
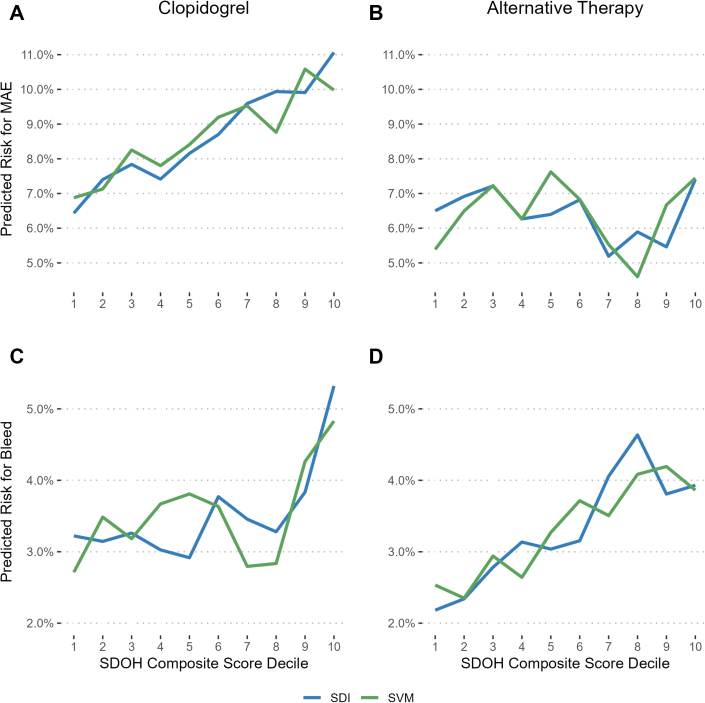
**Median Risk for Events by Social Determinants of Health Score Across Treatment Groups** Predicted risks were extracted from adjusted models for each outcome. Social determinants of health composite score deciles were determined based on the distribution of social determinants of health composite scores within the study cohort. Comparing model fit between linear and spline-based models revealed no significant changes in goodness-of-fit (likelihood ratio test *P* > 0.05). SDI = Social Deprivation Index; SVM = Social Vulnerability Metric; MAE = major adverse cardiovascular events; SDOH = social determinants of health.

A total of 261 (8.3%) patients had a MAE over a median follow-up of 352 (IQR: 178-362) days, corresponding to a rate of 11.6 (95% CI: 10.2-13.1) events per 100 PY. Among those receiving clopidogrel, MAE occurred in 172 (9.3%) patients at a rate of 13.6 (95% CI: 11.6-15.8) events per 100 PY, whereas MAE occurred in 89 (7%) of alternative therapy recipients rate: 9.0 (95% CI: 7.2-11.1 events per 100 PY) (Table 2). The associations between each score and MAE by treatment group are shown in Figure 2. The mean predicted risks by score decile for each treatment group are plotted in Figures 3A and 3B); no significant differences in goodness-of-fit were observed when comparing linear and spline-based models. Among clopidogrel-treated patients, each 1-unit increase in SDI score was associated with a significant increase in risk for MAE (unadjusted HR: 1.008; 95% CI: 1.002-1.015; *P* = 0.015), which persisted after adjusting for potential confounders (adjusted HR: 1.009; 95% CI: 1.001-1.016; *P* = 0.018). Unadjusted models showed a significant association between SVM percentile and risk for MAE in clopidogrel-treated patients (HR: 1.007; 95% CI: 1.002-1.013; *P* = 0.011), but the association was no longer statistically significant after adjustment (adjusted HR: 1.006; 95% CI: 1.000-1.012; *P* = 0.050). Among those on an alternative P2Y_12_ inhibitor, neither SDI nor SVM were associated with MAE risk (Figure 2).

**Table 2:** Incidence of Major Atherothrombotic Events and Clinically Significant Bleeding by Treatment Group

Treatment Group^a^	Major Atherothrombotic Events		Clinically Significant Bleeding	
	Event Count	Events per 100 PY (95% CI)	Event Count	Events per 100 PY (95% CI)
Overall cohort	261 (8.3%)	11.6 (10.2-13.1)	113 (3.6%)	5.0 (4.1-6.0)
Clopidogrel	172 (9.3%)	13.6 (11.6-15.8)	68 (3.7%)	5.4 (4.2-6.8)
Alternative therapy	89 (7%)	9.0 (7.2-11.1)	45 (3.4%)	4.4 (3.2-5.9)

Values are n (%) unless otherwise indicated.

PY = patient-years.

a Because of switches in P2Y12 inhibitor therapy and differential censoring by outcome of interest, the analysis cohort for clinically significant bleeding consisted of 1,829 clopidogrel-treated patients and 1,312 alternative therapy recipients.

### SDOH and bleeding outcomes

Clinically significant bleeding occurred in 113 (3.6%) of patients over a median follow-up of 353 (IQR: 180-362) days (rate of 5.0; 95% CI: 4.1-6.0 events per 100 PY; Table 2). Bleeding was observed in 68 (3.7%) clopidogrel-treated patients (rate of 5.4; 95% CI: 4.2-6.8 events per 100 PY) and in 45 (3.4%) of alternative therapy recipients (rate of 4.4; 95% CI: 3.2-5.9 events per 100 PY).

Among clopidogrel-treated patients, neither SDI nor SVM were associated with bleeding risk (Figure 2). Among those receiving alternative therapy, SDI was associated with an increased risk for bleeding on adjusted analysis (adjusted HR: 1.014; 95% CI: 1.001-1.028; *P* = 0.048), whereas there was no association between SVM and bleeding (adjusted HR: 1.009; 95% CI: 0.997-1.021; *P* = 0.143). The direction and magnitude of the associations observed in Cox models were consistent with those from Fine-Gray models, indicating that our findings were not significantly affected by the competing risk of death (Supplemental Table 2). The mean predicted risk for clinically significant bleeding by score decile for each treatment group is plotted in Figures 3C and 3D), with no significant differences in goodness-of-fit observed between linear and spline-based models.

### Clinical relevance of SDOH

**Figure 4:**
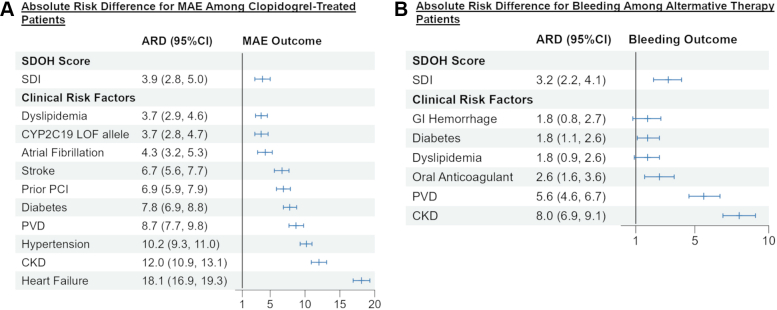
**Absolute Risk Difference in Outcomes per Clinical Risk Factor** Absolute risk difference for the Social Deprivation Index represents the risks associated with being in the top decile (most deprived) vs the bottom decile (least deprived) at 1 year post-PCI. MAE = major adverse cardiovascular events; SDOH = social determinants of health; ARD = absolute risk difference; CKD = chronic kidney disease; CYP2C19 = cytochrome P450 2C19; GI = gastrointestinal; LOF = loss-of-function; PVD = peripheral vascular disease; SDI = Social Deprivation Index; PCI = percutaneous coronary intervention.

Among clopidogrel-treated patients, the 1-year absolute risk difference for MAE between the highest (most deprived) and lowest (least deprived) SDI deciles was 3.9% (95% CI: 2.8%-5.0%) (Figure 4A). Among the established clinical predictors of MAE, the 1-year absolute risk difference ranged from 3.7% (95% CI: 2.9%-4.6%) for dyslipidemia to 18.1% (95% CI: 16.9%-19.3%) for heart failure. Among those receiving alternative therapy, the 1-year absolute risk difference for clinically significant bleeding between the highest and lowest SDI deciles was 3.2% (95% CI: 2.2%-4.1%) (Figure 4B), whereas the risk observed with a history of gastrointestinal bleeding was 1.8 (95% CI: 0.8-2.7), and the risk with oral anticoagulation was 2.6% (95% CI 1.6%-3.6%).

## Discussion

SDOH are nonmedical factors, including housing, income, and education, that influence health outcomes.^15^Although greater social deprivation has been associated with worse clinical outcomes following PCI, it remains unclear whether these risks differ by P2Y_12_ inhibitor therapy.^14^^,^^29^ In this real-world cohort of patients, we found that social deprivation was associated with increased risk for MAE following PCI among clopidogrel-treated patients (but not those on alternative therapy) and an increased risk for bleeding among those treated with alternative P2Y_12_ inhibitors (but not clopidogrel) (Central Illustration).

**Central Illustration:**
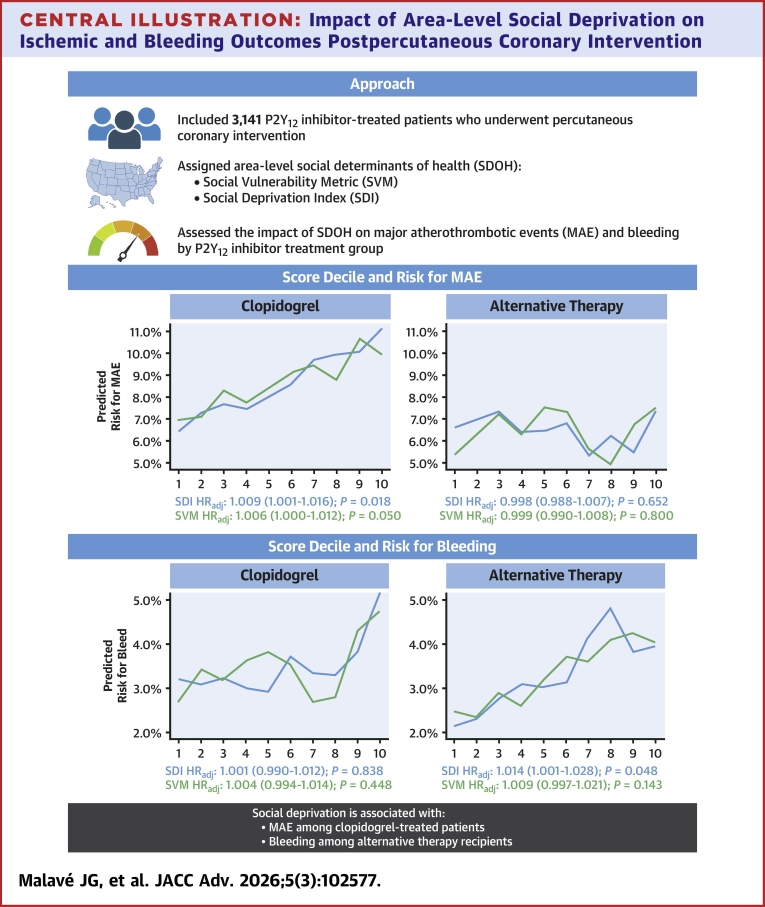
**Impact of Area-Level Social Deprivation on Ischemic and Bleeding Outcomes Postpercutaneous Coronary Intervention** Created in BioRender, Malave J (2026), https://BioRender.com/1oyc0wu. HRadj = adjusted HR.

To our knowledge, this is the first study to evaluate SDOH in relation to both ischemic and bleeding outcomes in a high atherothrombotic risk population who underwent PCI with clinical *CYP2C19* genotyping to guide P2Y_12_ inhibitor therapy. Prior studies examining SDOH in the context of PCI have predominantly focused on lower-risk populations, thereby underrepresenting the most vulnerable PCI patients,^29^ and none have assessed the impact of SDOH on bleeding, a key driver of P2Y_12_ inhibitor nonadherence and adverse cardiovascular events.^14^^,^^29^^,^^30^ In addition, previous analyses have not stratified outcomes by P2Y_12_ inhibitor type, despite well-documented variability in both ischemic and bleeding risk across P2Y_12_ inhibitors.^14^^,^^29^^,^^30^

### Clinical impact

Within our cohort, belonging to the highest vs lowest SDI decile was associated with an absolute risk difference comparable in magnitude to that of some established clinical risk factors for adverse outcomes. For ischemic outcomes among clopidogrel-treated patients, heart failure or chronic kidney disease were the major contributors to risk for events. However, patients in the top SDI decile experienced a risk for MAE similar to that observed with atrial fibrillation, dyslipidemia, or having a *CYP2C19* loss-of-function allele. For bleeding with alternative therapy, the risk attributed to SDI was comparable to that of anticoagulant use. These findings suggest that measures of SDOH are clinically meaningful risk factors, with effect sizes in line with traditional cardiovascular comorbidities routinely used to guide treatment decisions.

### Potential mechanism for SDOH contributing to clinical outcomes

Socioeconomic deprivation is associated with delayed care-seeking behavior.^31^ When combined with the inherent risk of each treatment group, variability in health care utilization across SDOH strata may contribute to observed differential risks. For example, alternative therapy recipients with reduced healthcare contact may have minor bleeds that went unreported until progressing to more severe bleeding requiring intervention. In contrast, clopidogrel-treated patients may have delayed seeking care for mild ischemic symptoms that eventually progressed to MAE. In this way, health-related behaviors may be important mediators in the association between area-level SDOH and clinical outcomes.

Another potential mechanism that may underlie the differential impact of social deprivation on outcomes by P2Y_12_ inhibitor type is related to glycemic control. Hyperglycemia is known to enhance platelet reactivity and activation.^32^ There is evidence that prasugrel is less effective at reducing platelet reactivity in insulin-dependent (but not noninsulin treated) diabetes, whereas ticagrelor consistently reduces platelet reactivity regardless of diabetes status.^33^ Clopidogrel-treated patients with diabetes are more likely to exhibit high platelet reactivity compared to nondiabetics, with more marked effects in insulin-treated patients.^34^^,^^35^ Moreover, good glycemic control (glycosylated hemoglobin ≤7%) is associated with a reduced risk of cardiovascular events among clopidogrel-treated patients.^36^ SDOH strongly influence diabetes management and medication access, with individuals from disadvantaged areas being more likely to have poor glycemic control.^37^ Thus, glycemic control may be an unmeasured confounder that disproportionately affects clopidogrel-treated patients in high-deprivation areas. However, this hypothesis requires testing.

Prasugrel and ticagrelor are associated with a higher risk of bleeding compared to clopidogrel.^8^ SDOH are closely linked to a greater burden of comorbidities, many of which are established predictors of bleeding, such as chronic kidney disease, uncontrolled hypertension, and cirrhosis.^38^, ^39^, ^40^, ^41^ While we did not capture data on liver function or hypertension control in our cohort, over 23% of patients had chronic kidney disease, and 11% were on oral anticoagulation, another well recognized risk factor for bleeding.^27^ In our analysis, higher social deprivation was associated with an increased risk of bleeding among patients receiving alternative therapy, but this association was not observed in those treated with clopidogrel. This disparity may reflect an additive effect of more potent P2Y_12_ inhibition combined with a higher comorbidity burden in socioeconomically disadvantaged populations.

### Differences between composite SDOH scores

We did not observe a significant association between SVM percentile and clinical outcomes, as we did with SDI, despite both being validated predictors of area-level mortality.^17^^,^^19^^,^^20^ This discrepancy may be explained by key differences in how each score is constructed. First, transportation is a well-established barrier to healthcare access, particularly among socioeconomically deprived populations. Lack of vehicle ownership, for example, has been linked to a 10% increased risk of mortality in patients with myocardial infarction.^42^ The SDI accounts for the percentage of households without a vehicle, while the SVM does not include any variables directly related to transportation access. Second, the SDI and SVM use different approaches to assess poverty. The SDI measures poverty as the percentage of individuals living below 100% of the federal poverty line, whereas the SVM uses median household income and the percentage of individuals with an income-to-poverty ratio between 1.25 and 1.99. In areas with substantial income inequality or socioeconomic diversity, the median income may obscure severe financial hardship. Additionally, the broader income range used by the SVM captures low-income individuals who may not meet the federal poverty threshold, potentially diluting its sensitivity to severe deprivation. Taken together, these differences suggest that the SDI may provide a more accurate representation of socioeconomic deprivation in our cohort, which has higher levels of social disadvantage compared to the general U.S. population.

### Health care disparities implications

From a health care disparities perspective, our findings highlight the importance of accounting for socioeconomic factors at the time of P2Y_12_ inhibitor selection. Although we are unable to establish a causal association between SDOH and the observed outcomes, individuals residing in socially deprived areas may benefit from closer monitoring and more intensive management of cardiovascular risk factors to reduce the risk for ischemic events in clopidogrel-treated patients and bleeding in those on alternative therapy. SDOH may complement traditional risk factors when selecting therapy that optimally balances ischemic and bleeding risk. Addressing socioeconomic disparities is paramount to ensuring healthcare equity and may require both individual-level interventions (eg, patient education and support services) as well as systemic changes (eg, improved access to care, and medication affordability).

### Study limitations

Our study has several limitations. SDI and SVM are measured at the ZCTA level, which may not capture more granular SDOH that influence clinical outcomes. As a result, within-area heterogeneity could mask important socioeconomic differences among individuals. Employing measures with higher geographic resolution (e.g., Area Deprivation Index) may influence the magnitude and direction of the observed associations.^43^ Additionally, SDOH composite scores were assigned based on the zip code of residence at the time of PCI. Individuals with misdocumented addresses or who relocated within 1 year of PCI are subject to misclassification. Additionally, SDOH composite scores do not account for rurality, access to health care, or individual socioeconomic factors. Furthermore, our study cohort presented with significantly higher social deprivation compared to the average U.S. population, limiting the generalizability of our findings to more affluent communities. However, similar SDI scores across our Florida and North Carolina study sites serve to enhance generalizability. Finally, treatment groups were assigned based on drug exposure at the time of event or last follow-up, as in previous analysis, introducing the potential for time-to-event overestimation in individuals who switch P2Y_12_ inhibitors during the follow-up period.^22^^,^^23^ Additional work comparing multiple area-level SDOH measures within a given population may strengthen our understanding of this association and help elucidate which factors most directly impact post-PCI outcomes.

## Conclusions

In summary, our findings showed that area-level social deprivation, as measured by the SDI score, was significantly associated with a higher risk for MAE in clopidogrel-treated patients and an increased risk for clinically significant bleeding in alternative therapy recipients, whereas the reverse associations were not observed. Future studies are needed to identify the specific clinical or socioeconomic factors driving these disparities to inform interventions to ameliorate worse outcomes in socially deprived individuals treated with P2Y_12_ inhibitor therapy following PCI.

## Funding support and author disclosures

This work was supported by grants from the 10.13039/100000002National Institutes of Health (NIH) 10.13039/100000050National Heart, Lung, and Blood Institute (R01HL149752) and 10.13039/100006108National Center for Advancing Translational Sciences (UM1TR004406). The content is solely the responsibility of the authors and does not necessarily represent the official views of the National Institutes of Health. Dr Shao is supported by 10.13039/100000002NIH grant T32GM086330 from the 10.13039/100000057National Institute of General Medical Sciences. Dr Malave is supported by 10.13039/100000002NIH grant T32HG008958 from the National Human Genomic Research Institute. Dr Franchi has received payment as an individual for consulting fee or honoraria from Werfen and institutional payments for grants from PLx Pharma and The Scott R. MacKenzie Foundation. Drs Cavallari and Lee have received research support from Werfen. Dr Angiolillo has received consulting fees or honoraria from Abbott, Amgen, Anthos, AstraZeneca, Bayer, Biosensors, Boehringer Ingelheim, Bristol-Myers Squibb, Chiesi, CSL Behring, Daiichi-Sankyo, Eli Lilly, Faraday, Haemonetics, Janssen, Merck, Novartis, Novo Nordisk, PhaseBio, PLx Pharma, Pfizer, and Sanofi. Dr Angiolillo's institution has received research grants from Amgen, AstraZeneca, Bayer, Biosensors, CeloNova, CSL Behring, Daiichi-Sankyo, Eisai, Eli Lilly, Gilead, Idorsia, Janssen, Matsutani Chemical Industry Co, Merck, Novartis, and the Scott R. MacKenzie Foundation. All other authors have reported that they have no relationships relevant to the contents of this paper to disclose.

## Figures and Tables

**JADV092925-1628_supp_BioRender_Subscription.png:**