# Supplementary material for: Impact of Area-Level Socioeconomic Deprivation on Post-PCI Outcomes Stratified by P2Y12 Inhibitor Therapy
Source: JACC Adv. 2026 Feb 3;5(3):102577. doi: 10.1016/j.jacadv.2025.102577 (PMC12892058; doi:10.1016/j.jacadv.2025.102577)
Supplement: Supplementary Table 1 [file mmc1.docx]

**Supplemental Appendix**

**Supplemental Table 1.** Variables comprising each area-level social determinants of health (SDOH) measure [^1,2^](https://sciwheel.com/work/citation?ids=13962972,17122883&pre=&pre=&suf=&suf=&sa=0,0&dbf=0&dbf=0)

| **SDOH Composite Score** | **SDOH Variables Considered** |
| --- | --- |
| **Social Vulnerability Metric (SVM)** | - Percentage of civilian veterans with a disability (ages 18–64) - Percentage of families with children that are single-parent families - Percentage of the population divorced or separated (ages 15 and over) - Percentage of children living with a grandparent householder (ages 17 and under) - Percentage of households with any internet connection - Percentage of households without a computer - Percentage of households with a smartphone with no other type of computing device - Percentage of employed working in finance and insurance, real estate, and rental and leasing - Percentage of employed working in professional, scientific, management, administrative, and waste management services - Percentage of population with income to poverty ratio: 1.25-1.99 - Median household income (in dollars, inflation-adjusted to file data year) - Percentage of population with a bachelor's degree (ages 25 and over) - Percentage of population with a master's or professional school degree or doctorate (ages 25 and over) - Percentage of population with only high school diploma (ages 25 and over) - Percentage of population with less than high school education (ages 25 and over) - Median home value of owner-occupied housing units - Percentage of housing units that are mobile homes - Percentage of housing units vacant - Convenience stores per 1,000 people - Percentage of population with any Medicaid/means-tested public health insurance coverage - Percentage of population with any private health insurance coverage - Percentage of population with employer-based health insurance - Percentage of population with Medicare, Medicaid, TRICARE/military, U.S. Department of Veterans Affairs (VA) coverage only - Percentage of population with no health insurance coverage |
| **Social Deprivation Index (SDI)** | - Percent population less than 100% federal poverty level - Percent population 25 years or more with less than 12 years of education - Percent non-employed for population 16-64 years - Percent households living in renter-occupied housing units - Percent households living in crowded housing units - Percent single parent families with dependents < 18 years - Percent households with no vehicle |

**Supplemental Table 2.** Fine–Gray Competing Risk Models for Bleeding With Death as a Competing Event Across Treatment Groups

| Treatment Group | Score | Unadjusted Subdistribution HR (95%CI) | *P* | Adjusted Subdistribution HR (95%CI) | *P* |
| --- | --- | --- | --- | --- | --- |
| Clopidogrel | SDI | 1.01 (0.995-1.02) | 0.240 | 1.002 (0.989-1.01) | 0.810 |
|  | SVM | 1.01 (0.996-1.01) | 0.240 | 1.00 (0.993-1.01) | 0.510 |
| Alternative Therapy | SDI | 1.01 (0.998-1.02) | 0.110 | 1.015 (1.000-1.03) | 0.05 |
|  | SVM | 1.01 (0.997-1.02) | 0.130 | 1.01 (0.997-1.02) | 0.120 |

**References**

[1.    Saulsberry L, Bhargava A, Zeng S, et al. The social vulnerability metric (SVM) as a new tool for public health. *Health Serv Res*. 2023;58(4):873-881. doi:10.1111/1475-6773.14102](https://sciwheel.com/work/bibliography/13962972)

[2.    Social deprivation index (SDI). Robert Graham Center - Policy Studies in Family Medicine & Primary Care. (2018, November 5). Retrieved November 29, 2021, from https://www.graham-center.org/rgc/maps-data-tools/sdi/social-deprivation-index.html.](https://sciwheel.com/work/bibliography/17122883)
